# Remifentanil-Sparing Effect of Pectoral Nerve Block Type II in Breast Surgery under Surgical Pleth Index-Guided Analgesia during Total Intravenous Anesthesia

**DOI:** 10.3390/jcm8081181

**Published:** 2019-08-07

**Authors:** Jung Ju Choi, Youn Yi Jo, Seung Hwan Kim, Wol Seon Jung, Dongchul Lee, Kwan Yeong Kim, Hyun Jeong Kwak

**Affiliations:** Department of Anesthesiology and Pain Medicine, Gachon University College of Medicine, Gil Hospital, Incheon 21565, Korea

**Keywords:** breast cancer surgery, post-operative pain, Pecs II block, opioid consumption

## Abstract

The pectoral nerve block type II (Pecs II block) can provide adequate perioperative analgesia in breast surgery. The surgical pleth index (SPI) is used to monitor the nociception balance using pulse oximetry. We investigated the remifentanil-sparing effect of Pecs II block under SPI guided analgesia during total intravenous anesthesia (TIVA). Thirty-nine patients undergoing breast surgery under remifentanil-propofol anesthesia were randomly assigned to the intervention (Pecs group, *n* = 20) or control group (*n* = 19). Remifentanil and propofol concentrations were adjusted to maintain an SPI of 20–50 and a bispectral index of 40–60, respectively. The Pecs group received an ultrasound-guided Pecs II block preoperatively using 30 mL of 0.5% ropivacaine. Total infused remifentanil during the surgery was significantly less in the Pecs group than in the control group (6.8 ± 2.2 μg/kg/h vs. 10.1 ± 3.7 μg/kg/h, *p* = 0.001). Pain scores on arrival at the postanesthetic care unit (PACU) (3 (2–5) vs. 5 (4–7)) and the rescue analgesic requirement in the PACU (9 vs. 2) was significantly lower in the Pecs group than in the control group. In conclusion, Pecs II block was able to reduce the intraoperative remifentanil consumption by approximately 30% and improve the postoperative pain in PACU in patients undergoing breast surgery under SPI-guided analgesia during TIVA.

## 1. Introduction

Pectoral nerve block (Pecs block) is a new technique for managing perioperative pain during breast surgery. While Pecs block type I (Pecs I block) is accomplished by injecting local anesthetics between the pectoralis major and minor muscles, Pecs block type II (Pecs II block) is an extension of Pecs I block that requires the additional injection of local anesthetics between the pectoralis minor and serratus anterior muscles [1,2]. The Pecs block rarely causes major complications such as spinal cord injury and epidural hematoma [3]. In recent studies, Pecs II block has been found to have perioperative analgesic effects during breast cancer surgery [4,5]. Further, since the Pecs II block blocks long thoracic and thoracodorsal nerves in addition to the lateral branches of the intercostal nerves that innervate the mammary gland and the skin from the 2nd to 6th thoracic dermatomes, it can also be useful in mastectomy with axillary clearance [1]. A previous clinical study demonstrated that Pecs II block reduced the amount of intraoperative fentanyl required and the postoperative pain control, in this case, morphine requirement [6]. A retrospective analysis of 146 patients also reported that the cumulative distribution of remifentanil requirement in patients undergoing breast surgery was significantly lower in patients who received Pecs II block than in those who did not [7].

Because total intravenous anesthesia (TIVA) has less risk of postoperative nausea and vomiting (PONV) than inhalation anesthesia, it is often the preferred choice for patients with a high risk for PONV [8]. Among the many opioids that are currently available, remifentanil is characterized by its potent, rapid onset and the predictability of the offset, even after a long infusion period, without accumulation. Thus, remifentanil is a preferred choice during the TIVA anesthetic method [9]. However, if it is used in high concentrations intraoperatively, it increases postoperative pain and the rescue analgesic requirement due to its acute tolerance [10].

The surgical pleth index (SPI) is a non-invasive, dimensionless score (0–100) that allows for the estimation of intraoperative nociception, and it can also easily monitor the nociception–anti-nociception balance by using pulse oximetry during general anesthesia [11]. Pulse plethysmography, which correlates with the balance of the autonomic nervous system, and photo-plethysmography from pulse oximetry data determine the SPI [11]. The SPI has been reported to be correlated with surgical stimulation and the effect-site concentration of remifentanil [12]. A previous study of 170 outpatients showed that remifentanil dose adjustments guided by the SPI significantly reduced the opioid requirement compared with that in the non-SPI monitored group [13]. A recent meta-analysis also demonstrated that SPI-guided opioid adjustment might help to reduce the intraoperative dosage of opioids [14].

We hypothesized that Pecs II block might be effective in reducing the intraoperative remifentanil requirement in SPI-guided analgesia during TIVA, and so, would lead to improved postoperative analgesia. Therefore, we evaluated the remifentanil-sparing effect of Pecs II block in SPI-guided analgesia during TIVA for breast conserving surgery (BCS) with sentinel lymph node biopsy (SLNB).

## 2. Materials and Methods

The ethics committee of Gachon University Gil Hospital approved this study. The study was registered at www.ClinicalTrials.gov (NCT03210220) prior to patient recruitment. Written informed consent was provided by all participants.

The inclusion criteria for this study were the following: women, aged between 20 and 65 years, with an American Society of Anesthesiologists physical status of 1 or 2, who were scheduled for BCS with SLNB for the treatment of breast cancer. The exclusion criteria were as follows: patients receiving anticoagulant therapy, those with bleeding disorders, hypersensitivity to local anesthetics, body mass index greater than 35 kg/m^2^, the presence of spine or chest wall deformities, and pregnancy. Patients either received the Pecs II block (Pecs group) or did not receive it (control group). One anesthesiologist (JJC) performed all blocks in the Pecs group enrolled patients. After the intervention, the participants and the investigator responsible for the study outcome assessment were blinded.

Premedication was not given. Electrocardiography, non-invasive blood pressure monitoring and the bispectral index (BIS vista monitor revision 3.0; Aspect Medical Systems, Norwood, MA, USA) were applied in the operating room. SPI (S5 monitor; GE Healthcare, Helsinki, Finland) was monitored using a pulse oximeter sensor attached to the index finger contralateral to the operative site. For anesthesia induction and maintenance, lidocaine (1 mg/kg), propofol, and remifentanil were administered. The effect-site concentrations of propofol and remifentanil were automatically calculated by Schnider’s [15] and Minto’s [16] pharmacokinetic models, respectively, using a target-controlled infusion (TCI) pump (Orchestra; Fresenius Kabi, Bad Homburg, Germany). To facilitate orotracheal intubation, rocuronium (0.8 mg/kg) was administered after loss of consciousness.

For the patients in the Pecs group, a linear ultrasound probe was placed on the lateral third of the clavicle with bilateral abduction in the supine position. After identifying the axillary vein and artery, the ultrasound probe was positioned inferio-laterally, between the 3rd and 4th ribs, and then the pectoralis major and minor, and serratus anterior muscles were confirmed. The needle was advanced in a medio-lateral direction in-plane view of the ultrasound. For the Pecs II block, a total 30 mL of 0.5% ropivacaine was injected. First, the needle tip was advanced into the fascia between the pectoralis major and minor muscles and 10 mL of 0.5% ropivacaine was injected. Thereafter, the needle tip was advanced into the tissue plane between the pectoralis minor and serratus anterior muscles, and 20 mL of 0.5% ropivacaine was injected in a similar manner.

Fifteen minutes after the Pecs II block, a skin incision was made for the scheduled surgery. During the surgery, the remifentanil dose was adjusted to a target SPI of 20–50 and the propofol dose was adjusted to a target BIS of 40–60, using a TCI pump. When the SPI was >50 or <20, the remifentanil effect-site concentration was adjusted to a step of 0.5 ng/kg with intervals of 1 min or more. When the BIS value was >60 or <40, the propofol effect-site concentration was adjusted to a step of 0.5 µg/kg with intervals of 1 min or more. When the systolic blood pressure dropped below 90 mm Hg or below 80% of the baseline value, ephedrine 5 mg was administered intravenously at 2-min intervals. If the heart rate (HR) dropped below 50 beats/min, atropine 0.5 mg was administered intravenously.

Patient-controlled analgesia (PCA) (Accufuser plus^®^, Wooyoung medical, Seoul, Korea) was provided for 48 h with ketorolac 120 mg and sufentanil 50 µg in normal saline 100 mL, (basal infusion rate 2 mL/h, 0.5 mL intermittent bolus with a 15 min lock-out interval). For preventing PONV, ramosetron 0.3 mg was administered intravenously before the end of the surgery.

In the postanesthetic care unit (PACU), the postoperative pain was evaluated using an 11-point numerical rating scale (NRS) (0–10) and was accessed immediately after arrival at the PACU, 1 h postoperatively, 16–24 h postoperatively, and 24–48 h postoperatively. Fentanyl 50 µg bolus was administered as a rescue analgesic agent when the NRS was greater than 5 points.

The primary outcome was the intraoperative remifentanil consumption, and the secondary outcomes were the postoperative pain score and rescue analgesic requirement. The sample size was calculated based on the previous study of breast cancer surgery under TIVA [7]. The intraoperative remifentanil consumption was 10.9 ± 2.9 μg/kg/h and 7.3 ± 3.3 μg/kg/h in the control and Pecs groups, respectively [7]. With type 1 error of 0.05 and a power of 0.9, the study required 14 patients in each group. Considering the possible drop-out, 20 patients were included in each group. Patients were randomly assigned to the Pecs group (*n* = 20) or the control group (*n* = 20), based on a randomized list generated with Excel 2013 (Microsoft Office, Redmond, WA, USA), without stratification.

Data were analyzed using SPSS 19.0 (SPSS, Chicago, IL, USA). Values were presented as number of patients, medians (interquartile ranges; IQR) or mean ± standard deviation (SD). The Kolmogorov-Smirnov test was performed for the normality test of continuous variables. The normally distributed data were presented as mean ± SD and the skewed data were presented as median (IQR). An independent *t*-test was used for normally distributed variables (intraoperative dosages of remifentanil and propofol), and the Mann–Whitney U Test was used for variables with a non-normal distribution (postoperative pain scores). The chi-squared test or Fisher’s exact test was used to analyze the categorical data (the use of atropine, ephedrine and rescue analgesics), where appropriate. Repeated, measured ANOVA was used for accessing the intergroup differences of MBP, HR, SPI, and BIS over time. Statistical significance was accepted for *p* value < 0.05.

## 3. Results

**Figure 1:**
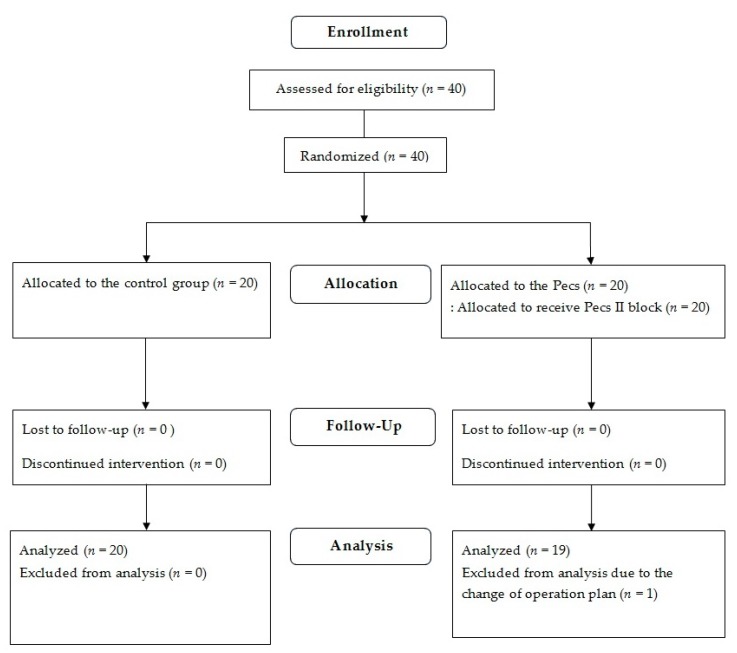
Flow diagram of patient allocation.

Recruitment of the patients took place from August 2017 to September 2018. Of 40 initially enrolled patients, one patient in the Pecs group was excluded from the analysis due to a change of surgical plan (Figure 1).

The patients’ characteristics are shown in Table 1. Age, weight, and height were not significantly different between the two groups. Surgery and anesthesia time were not different between the two groups.

The perioperative clinical data are summarized in Table 2. The infused dosage of remifentanil was higher in the control group than in the Pecs group (10.1 ± 3.7 µg/kg/h versus 6.8 ± 2.2 µg/kg/h, respectively; *p* = 0.001), while that of propofol was similar in both the groups (6.4 ± 1.1 mg/kg/h versus 6.2 ± 1.1 mg/kg/h, respectively; *p* = 0.515). The intraoperative requirements for atropine and ephedrine were similar for both groups. MBP and HR measured immediately on arrival at the PACU and after 1 h in the PACU showed no differences between the groups. The pain score (median (interquartile range)) was significantly lower in the Pecs group than in the control group upon arrival at the PACU (5 (4–7) versus 3 (2–5), *p* = 0.001) and at 1 h after PACU arrival (4 (2.25–5.75) vs. 3 (2–3), *p* = 0.041). The number of patients requiring rescue analgesics while in the PACU was significantly lower in the Pecs group than in the control group (9 versus 2, *p* = 0.017). None of the patients in either group required rescue antiemetics while in the PACU. The postoperative pain scores were similar in the two groups on postoperative day (POD) 1 and 2.

**Figure 2:**
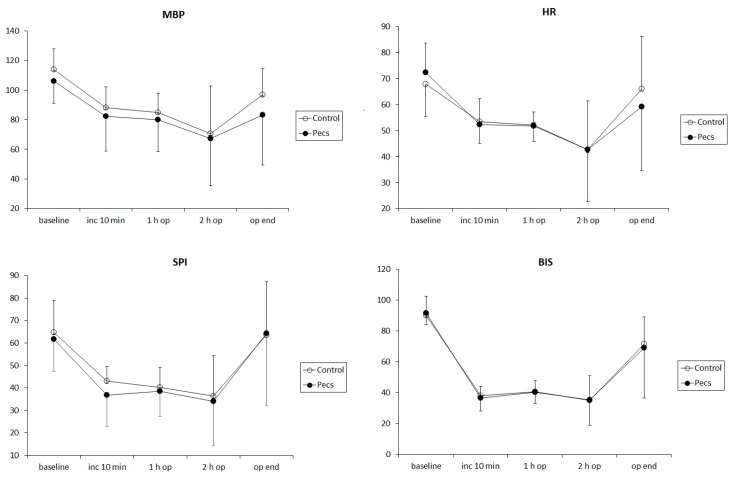
Intraoperative changes in the mean blood pressure (MBP, left upper), heart rate (HR, right upper), surgical pleth index (SPI, left lower) and bispectral index (BIS, right lower). Error bars mean standard deviations of each value. Data were measured at 5 time points: at baseline, 10 min (inc 10 min), 1 h (1 h op), and 2 h (2 h op) after the surgical incision, and at the end of surgery (op end), respectively. There were no intergroup differences in the changes in MBP, HR, SPI, and BIS during the surgery. The patients either received a pectoral nerve block type II (Pecs group, filled circle) or not (control group, unfilled circle).

The intraoperative changes in mean blood pressure (MBP), HR, SPI, and BIS are illustrated in Figure 2. The changes in MBP, HR, SPI, and BIS were significant over time (*p* = 0.002, <0.001, <0.001, and <0.001, respectively) without intergroup differences (*p* = 0.591, 0.681, 0.371, and 0.662, respectively).

## 4. Discussion

In this prospective randomized study, Pecs II block significantly reduced the remifentanil requirement under SPI-guided analgesia during TIVA for BCS with SLNB, as well as the postoperative pain score, with a lesser requirement of rescue analgesics in the PACU. To the best of our knowledge, this is the first study that has demonstrated the opioid-sparing effect of Pecs II block under SPI-guided analgesia during TIVA for breast cancer surgery.

During surgery, excessive nociceptive stimulation is known to affect the length of hospital stay, overall costs of hospital care, and patient outcomes [17,18]. Although electroencephalography (EEG)-based hypnosis monitors, such as BIS, might reduce the dose of hypnotic agents and achieve hemodynamic stability with reduced intraoperative awareness [19], BIS might not be suitable for measuring the response to noxious stimuli [20]. BIS is known to be able to predict loss of response to verbal command, but not to noxious stimuli [20]. The differences in the brain regions for analgesia and hypnosis might influence the poor response to noxious stimuli of the EEG-based monitoring devices in the frontal cortex [13].

SPI is a normalized score (0–100), which is calculated from the amplitude of a photoplethysmographic pulse wave and the heart-beat interval. SPI scores are superior predictors of intraoperative analgesia, compared to entropy variables, heart rate or photoplethysmographic pulse wave amplitude, alone [21]. An earlier clinical study showed that SPI was closely correlated with the effect-site concentration of remifentanil compared to the response entropy, state entropy, and heart rate, and thus concluded that SPI appeared to be a good measure for maintaining nociception–anti-nociception balance [12]. SPI-guided analgesia has been demonstrated to reduce intraoperative opioid consumption and extubation time [22]. This is consistent with our results. In this study, during SPI-guided analgesia and BIS-guided sedation, Pecs II block reduced the intraoperative remifentanil consumption, but not the propofol consumption. In contrast, in a study using only BIS-guided TIVA, Pecs block has been reported to decrease the propofol requirement, but not the remifentanil requirement [23].

A commonly used technique for postoperative analgesia in breast surgery has been the thoracic paravertebral block. However, it does not cover the innervation area of the medial and lateral pectoral nerves, and therefore this leads to inadequate analgesia in the axillary and upper limb after breast surgery [4]. Conversely, the ultrasound-guided Pecs II block is an inter-fascial plane block, whereby local anesthetics are administered between the serratus anterior and the pectoralis minor muscles in the interspace of the pectoralis major and minor muscles. Thus, it blocks the long thoracic nerve, intercostobrachial nerve, pectoral nerve, and intercostal 3rd, 4th, 5th, and 6th nerves, leading to better pain relief in mastectomy with axillary clearance [1]. In addition, while thoracic paravertebral block carries the risk of accidental nerve injury, pneumothorax, vascular puncture, and sympathetic block [24,25], the ultrasound-guided Pecs II block has much less risk of such complications. A comparative study proved that Pecs II block had a significantly longer analgesic effect compared to that of the paravertebral block (294 ± 53 min vs. 198 ± 31 min) in patients undergoing radical mastectomy [4]. Furthermore, the requirement of 24-h morphine was significantly less with the Pecs II block compared to the paravertebral block (3.90 ± 0.79 vs. 5.30 ± 0.98 mg), and the pain score was lower during the postoperative 2 h [4].

The report of Morioka et al. [7] analyzing 146 cases of BCS showed that Pecs block reduced the remifentanil dosage by about 33% compared to the non-Pecs group [7]. Another clinical study of 80 breast cancer surgeries reported that the Pecs II block significantly reduced the opioid requirements compared to the control group, but it did not obtain significant difference in the frequency of postoperative rescue analgesics [5]. In this study, we found that the intraoperative remifentanil consumption was significantly decreased, by about 30%, and the postoperative pain scores and the use of rescue analgesics in the PACU were also decreased. Furthermore, the lower postoperative pain score in the Pecs group can be explained by the reduced acute opioid tolerance or opioid-induced hyperalgesia due to the lower dose of intraoperative remifentanil, in addition to the analgesic effect of Pecs II block itself [26].

There are some limitations in our study. First, since the blocks were performed after the anesthetic induction in an unconscious state, we were not able to precisely measure the success and strength of the block. However, we directly confirmed the spread of the local anesthetic under the ultrasound monitor and confirmed that there were no side effects, such as pneumothorax. Second, we were not able to follow-up the long-term effects of the preoperative Pecs II block. According to a survey of 3754 patients who underwent breast cancer surgery, 47% of the patients complained of persistent pain, and this pain was associated with the adjuvant radiotherapy, young age, and axillary lymph node dissection [27]. In our study, we demonstrated that the preoperative Pecs II block may mitigate the acute postoperative pain, and it can also be expected to reduce the number of patients who continue to have chronic pain after the surgery. A long-term follow-up might be helpful in revealing the value of the preoperative Pecs II block for breast cancer surgery.

## 5. Conclusions

In conclusion, our randomized controlled study suggested that pre-emptive Pecs II block reduced the intraoperative remifentanil consumption by 30% and improved the acute postoperative pain in patients with BCS and SLNB under SPI-guided analgesia during TIVA.

## Figures and Tables

**Table 1:** Patients’ characteristics.

Variables	Control (*n* = 20)	Pecs (*n* = 19)	Standardized Differences
Age (year)	52.7 ± 9.2	51.4 ± 7.7	2.7 (−4.2–6.8)
Weight (kg)	63.5 ± 8.0	59.4 ± 10.0	4.1 (−1.8–9.9)
Height (cm)	159.1 ± 5.7	158.8 ± 6.3	0.3 (−3.7–4.2)

Values are presented as mean ± SD or mean (95% confidence intervals). Control group: Patients without nerve block, Pecs block: Patients with pectoral nerve block type II before the surgery.

**Table 2:** Perioperative clinical data.

Variables	Control (*n* = 20)	Pecs (*n* = 19)	*p*-Value
During surgery			
Remifentanil consumption (μg/kg/h)	10.1 ± 3.7	6.8 ± 2.2	0.001
Propofol consumption (mg/kg/h)	6.4 ± 1.1	6.2 ± 1.1	0.515
Use of atropine (*n*)	5	2	0.239
Use of ephedrine (*n*)	6	6	0.915
Upon arrival at the PACU			
MBP (mmHg)	103 ± 18	96 ± 5	0.210
HR (beats/min)	76 ± 17	77 ± 10	0.830
Pain score (NRS)	5 (4–7 (2–8))	3 (2–5 (0–6))	0.001
After 1 h in the PACU			
MBP (mmHg)	106 ± 17	100 ± 20	0.310
HR (beats/min)	66 ± 13	69 ± 10	0.478
Pain score (NRS)	4 (2.25–5.75 (0–8))	3 (2–3 (1–5))	0.041
Rescue fentanyl requirement (n)	9	2	0.017
POD 1			
Pain score (NRS)	3 (2–3 (0–6))	2 (1–3 (0–7))	0.236
POD 2			
Pain score (NRS)	1 (1–2 (0–3))	1 (1–2 (0–4))	0.681

Values are presented as mean ± standard deviation, number of patients or median (IQR (range)). Control group, patients without nerve block; Pecs block, Patients with pectoral nerve block type II before the surgery; PACU, postanesthetic care unit; NRS, 11-points numeric rating scale (0–10); POD, postoperative day.
